# Development of Graphene-Based Polymeric Nanocomposites: A Brief Overview

**DOI:** 10.3390/polym13172978

**Published:** 2021-09-02

**Authors:** Ana M. Díez-Pascual

**Affiliations:** Universidad de Alcalá, Facultad de Ciencias, Departamento de Química Analítica, Química Física e Ingeniería Química, Ctra. Madrid-Barcelona, Km. 33.6, 28805 Alcalá de Henares, Madrid, Spain; am.diez@uah.es; Tel.: +34-918-856-430

**Keywords:** polymer nanocomposites, graphene, graphene oxide, functionalization, synergistic effects, energy storage

## Abstract

Graphene (G) and its derivatives, such as graphene oxide (GO) and reduced GO (rGO), have outstanding electrical, mechanical, thermal, optical, and electrochemical properties, owed to their 2D structure and large specific surface area. Further, their combination with polymers leads to novel nanocomposites with enhanced structural and functional properties due to synergistic effects. Such nanocomposites are becoming increasingly useful in a wide variety of fields ranging from biomedicine to the electronics and energy storage applications. In this review, a brief introduction on the aforementioned G derivatives is presented, and different strategies to develop polymeric nanocomposites are described. Several functionalization methods including covalent and non-covalent approaches to increase their interaction with polymers are summarized, and selected examples are provided. Further, applications of this type of nanocomposites in the field of energy are discussed, including lithium-ion batteries, supercapacitors, transparent conductive electrodes, counter electrodes of dye-sensitized solar cells, and active layers of organic solar cells. Finally, the challenges and future outlook for G-based polymeric nanocomposites are discussed.

## 1. Introduction

Reinforced polymers contain a polymeric matrix and a rigid filler that experiences a drastic change in modulus or stress at a given strain over the pure polymer. Conventional fillers include glass fibers, carbon fibers, carbon black, silica, calcium carbonate particles, and so forth, in the micrometer range. Though, most micron sized fillers need high loadings to attain moderate property improvements, leading to melt flow and processing issues owed to the high viscosity of the filled materials [1]. Besides, the high density of some conventional fillers frequently results in heavy composites. Furthermore, the poor interfacial interaction between the filler and the polymeric matrix leads to weak interfacial adhesion and results in composite failure.

A wide variety of filler sizes has been used for reinforcing polymeric matrices [2], and the best performance has been detected in the particle size range of 100 nm and below. Nanofillers in the range of 3–5 wt% attain comparable reinforcement as 20–30 wt% of microsized fillers [3]. Therefore, nanocomposites have a weight advantage over conventional composites and, nanoscale materials have emerged as suitable fillers owed to their increased specific interfacial area that enables stronger interfacial interactions and henceforth, superior modulus [4,5].

According to their dimensions, nanofillers can be classified into 1D, that include nanotubes and nanowires, 2D such as nanoclays and graphene (G), and 3D such as spherical nanoparticles. Amongst them, G is one of the most widely studied over the last decade due to its exceptional combination of electrical, mechanical, thermal, optical, and electrochemical properties. In particular, it is a promising nanomaterial for applications in various energy-related fields such as supercapacitors [6], batteries [7], solar cells [8], and fuel cells [9]. For such goal, G-based nanomaterials are frequently blended with polymers to form functional composites [10]. The polymer can enhance either the processability, flexibility, or both, of graphene materials, and also offer them new functions. To date, a huge number of G-based nanocomposites with insulating or conducting polymers have been prepared through noncovalent or covalent approaches [11,12].

This review focuses on summarizing the recent advances in the synthesis of polymer/G nanocomposites. Covalent and non-covalent functionalization methods are summarized, and selected examples are provided. Furthermore, applications of this type of nanocomposites in the field of energy are described, such as lithium-ion batteries, supercapacitors, transparent conductive electrodes, counter electrodes of dye-sensitized solar cells, organic solar cells, and so forth. Finally, the challenges and future perspectives for G-based polymeric nanocomposites are discussed.

## 2. Graphene and Its Derivatives: Synthesis and Characteristics 

### 2.1. Properties and Synthesis Methods of Graphene 

**Scheme 1:**
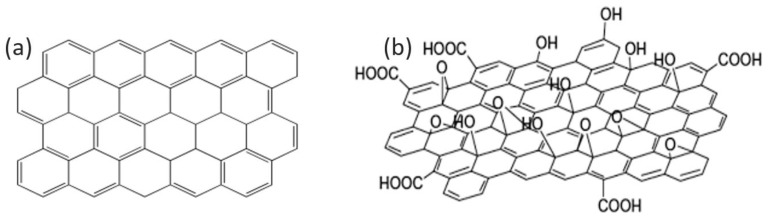
Chemical structure of graphene (**a**) and graphene oxide (**b**).

G is a 2D flat single-atom-thick sheet composed of sp^2^ carbon structure arranged in a honeycomb structure (Scheme 1a). G exhibits exceptional electronic, thermal, and mechanical properties [13]. It presents high gas impermeability, a high specific area (2600 m^2^/g), larger than that of carbon nanotubes (about 1000 m^2^/g), and a delocalized movement of electrons, which implies an outstanding electronic behavior and very high electron mobility (i.e., 15,000 cm^2^/V s) [14]. It displays very high electrical conductivity (6000 S/cm) and thermal conductivity (between 4800 and 5300 W/m K at room temperature) [15], significantly higher than that of Cu (400 W/m K). Besides, it is believed to be the thinnest material on earth and the strongest in terms of stiffness and strength, with a breaking strength 200 times greater than steel, a tensile strength of 130 GPa, and a Young’s modulus close to 1 TPa [16]. On the other hand, it shows high optical transparency [17], it is chemically inert, and electrochemically stable. Due to these properties, G is regarded as an excellent candidate for a variety of applications: (1) sensing and biosensing devices; (2) ultracapacitors that could store more renewable energy from sun, wind, and waves than current technologies; (3) transparent conducting electrodes required in applications such as touch screens, liquid crystal displays, organic photovoltaic cells; (4) light emitting diodes (LEDs); and (5) transistors and microchips or nanochips with high operating speed.

**Scheme 2:**
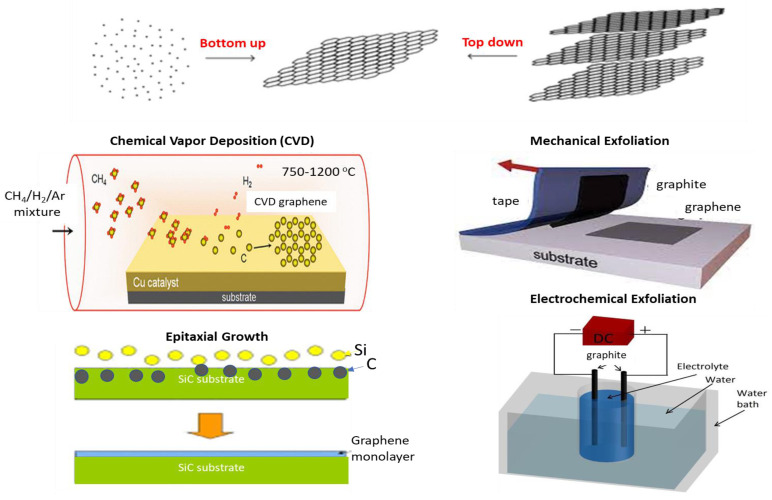
Schematic representation of the bottom-up and top-down approaches for graphene synthesis.

The synthesis of G can be carried out following two different approaches: (1) bottom-up method; and (2) top-down method. The former approach is based on the synthesis of G from small units of carbon to obtain graphene layers. The most common techniques based on this methodology are chemical vapor deposition (CVD) and epitaxial growth (Scheme 2).

A brief description of these methods along with their advantages and limitations are shown below.

CVD produces high-quality G using a transition metal substrate such as Cu, Ni, Pd, Au, or Ru [18]. The deposition consists of the flow of a hydrocarbon precursor at high temperature (i.e., 750–1200 °C) (Scheme 2). As the surface of the metal cools, a deposition phenomenon takes place due to the reduction in the solubility of carbon atoms, which precipitate forming a monolayer. Thus, CVD graphene is synthesized via two stages: (1) the pyrolytic decomposition of precursors, which is performed at very high temperatures with the aid of a metal catalyst and onto a substrate to prevent the precipitation of carbon clusters; and (2) the formation of G monolayer out of the disassociated carbon atoms, which also requires very high temperatures, hence catalysts are employed. This method is scalable and enables the synthesis of large graphene flakes. The main disadvantage is that the catalysts can incorporate impurities into G monolayer.

Epitaxial growth is usually carried out using a silicon carbide (SiC) substrate [19]. The substrate is graphitized under thermal treatment at ~1300 °C and vacuum conditions, which leads to the sublimation of the Si atoms at the same time as the carbon-enriched surface experiences reorganization and graphitization. This technique allows controlling of the G layer thickness via modifying either the time, the temperature, or in combination, and enables obtaining high-quality G. One of its advantages is that it enables electric devices to be fabricated directly on semi-insulating SiC [20]. Though, it is one of the most expensive synthesis methods given that SiC has to be heated at very high temperatures; thus, it is not reasonably priced on a large-scale.

Regarding the top-down methods (Scheme 2), the most widely employed are mechanical exfoliation and the electrochemical exfoliation. The former is the method developed by Andre Geim and Konstantin Novoselov in 2004 [21] to isolate G by peeling it off from graphite flakes using Scotch tape. Since the interlayer van der Waals forces in graphite are very weak (interaction energy of ~2 eV/nm), graphite can be easily exfoliated using an adhesive tape or atomic force microscopy (AFM). This method yields good quality G sheets; however, it is not suitable for mass production. G can also be exfoliated in liquid media, which uses specific organic molecules or surfactants that intercalate between the G sheets. 

Another widely used process is electrochemical exfoliation [22], based on the penetration of graphite by ions from the solution forced by the applied potential. G obtained by this approach can be dispersed in organic solvents such as DMF, which enables the fabrication of thin films. By modifying a number of parameters such as the voltage applied, the time, and concentration of the electrolyte, the properties of the resulting layer can be tailored. This is a simple and cheap method, that could easily be scaled up. 

### 2.2. Characteristics and Synthesis Methods of Graphene Oxide

Graphene oxide (GO) is an oxidized layer of G that contains epoxides, hydroxyls, and carbonyls on the basal planes and carboxylic acids on the edges (Scheme 1). Consequently, some properties of GO differ from those of G: The sp^3^ carbon atoms in GO increase the interlayer spacing, improving its ability to hold compounds. The functional groups also modify the electronic structure; thus its electronic properties are worse than those of pristine G (i.e., lower electron mobility and lower conductivity, it is typically insulating with a sheet resistance value of around 1000 Ω/sq). It presents aqueous processability, surface functionalization capability, amphiphilicity, biocompatibility, and ability to interact with biological cells and tissues [23]. It is impermeable to gases and vapors except for water, hence it can be employed in membranes. Further, it can form stable aqueous dispersions by simple and cheap sonication processes, which is critical for large-scale uses. 

**Scheme 3:**
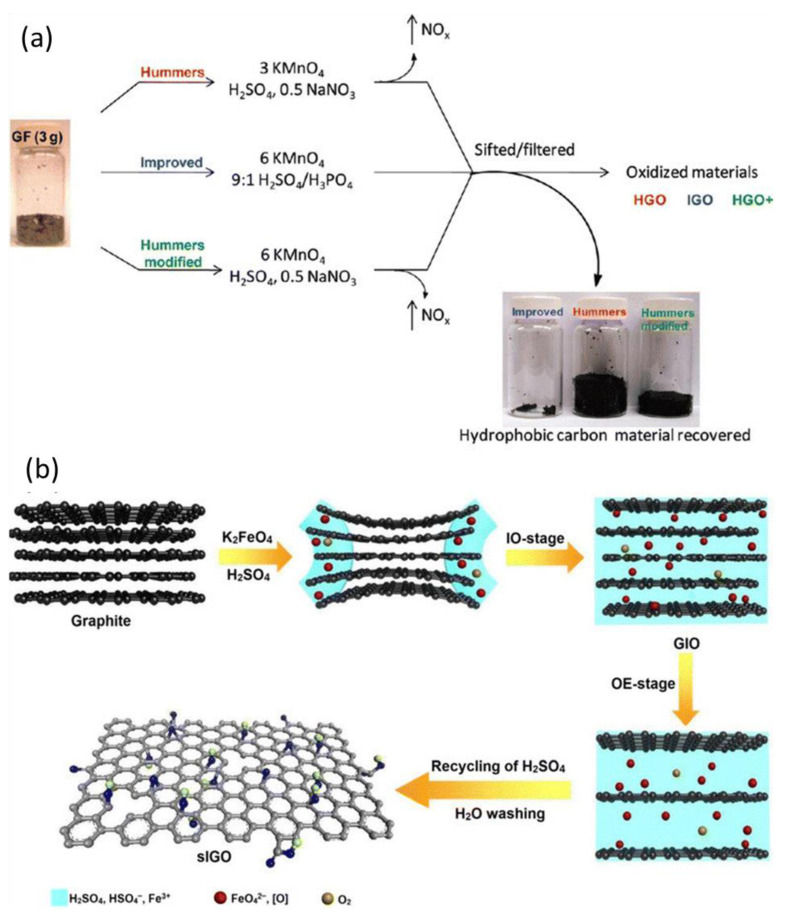
(**a**): Oxidation of graphite flakes (GF) by Hummer’s method, improved Hummer’s method, and modified Hummer’s method (**b**). Synthesis of single layer graphene oxide (slGO) by K_2_FeO_4_. Reprinted with permission from Ref. [26]. Copyright 2015 Creative Commons License.

Different methods to prepare GO have been reported [24]: Graphite oxide was synthesized for the first time about 150 years ago by Brodie using KClO_3_ and HNO_3_. This approach was improved by Staudenmaier in 1898 and in 1937 by Hofmann who used concentrated H_2_SO_4_, HNO_3_, and KClO_3_ to generate highly oxidized graphite. Though, this method was time-consuming (nearly 1 week) and harmful since toxic gases are generated (ClO_2_ and NO_x_). In 1958, Hummers reported a novel procedure by replacing HNO_3_ and KClO_3_ with NaNO_3_ and KMnO_4_ (Scheme 3a), and this has been the most widely employed since the first exfoliation of G in 2004. The C/O ratio of the GO synthesized is in the range of 1–2.9. The contaminants are commonly ash and water, though toxic gases such as N_2_O_4_ and NO_2_ can evolve. Hummer’s method has been improved using a 9:1 mixture of concentrated H_2_SO_4_/H_3_PO_4_ and KMnO_4_ to achieve high amounts of GO and less toxic gases in a short period [25] (Scheme 3a). Many researchers have focused on improving the Hummers’ method to make it either more efficient, environmentally friendly, or both. One modification is the addition of extra KMnO_4_ [26] (Scheme 3a) to attain higher oxidation efficiency. Other approaches try to avoid the production of toxic gases via replacing NaNO_3_ with other oxidants. In particular, the addition of persulfate (S_2_O_8_^2−^) ensures the complete oxidation and exfoliation of graphite [27]. To further increase the oxidation efficiency without heavy metal and toxic gases, K_2_FeO_4_ can be mixed with concentrated H_2_SO_4_, and GO can be synthesized in 1 h at room temperature via a two-stage process (Scheme 3b): in the intercalation oxidation (IO) stage, the in situ formed oxidants (FeO_4_^2−^ and atomic oxygen) and O_2_ intercalate into graphite layers and form intercalated graphite oxide (GIO). Further, in an oxidation-exfoliation (OE) stage, it is further oxidized and exfoliated by O_2_, and slGO is obtained [28]. Another path to produce GO is the sonication, stirring (or a combination of both) of graphite oxide in various solvents, such as water, dimethyl formamide (DMF), tetrahydrofuran (THF), and chloroform [29]. Sonication is a time-effective way of fully exfoliating graphite oxide, although it can seriously harm the graphene flakes, reducing their size from microns to nanometers, and even producing graphene platelets. Mechanically stirring is a less heavy-handed approach, albeit involving longer periods of time. Synergistic effect between intercalation (oxidation) and ultrasonication have also been reported [30]; this method requires less acid, is time saving/less energy consuming, offers higher productivity, and releases fewer toxic gases than the Hummers’ method. Monolayers of GO with layer spacing of ∼1 nm can be obtained in less than 1 h. Besides, this procedure is safe and can be applicable for large scale manufacture of GO. Eco-friendly protocols for large-scale production of GO using citric acid have also been reported [31], which preserved yield, and avoided extensive sonication and toxic gas evolution, with promising applications in composites, energy storage, and reinforcement, where a large quantity of oxidized graphene is needed. 

### 2.3. Synthesis and Characteristics of Reduced Graphene Oxide

The reduced GO (rGO) sheets are obtained via elimination of functional groups from GO. The goal is to attain graphene-like materials analogous to the pristine graphene. Though, residual functional groups and defects substantially change the structure of the carbon plane, consequently, the characteristics of rGO vary from those of G. So, the electrical conductivity of rGO is generally several orders of magnitude lower than that of G.

**Scheme 4:**
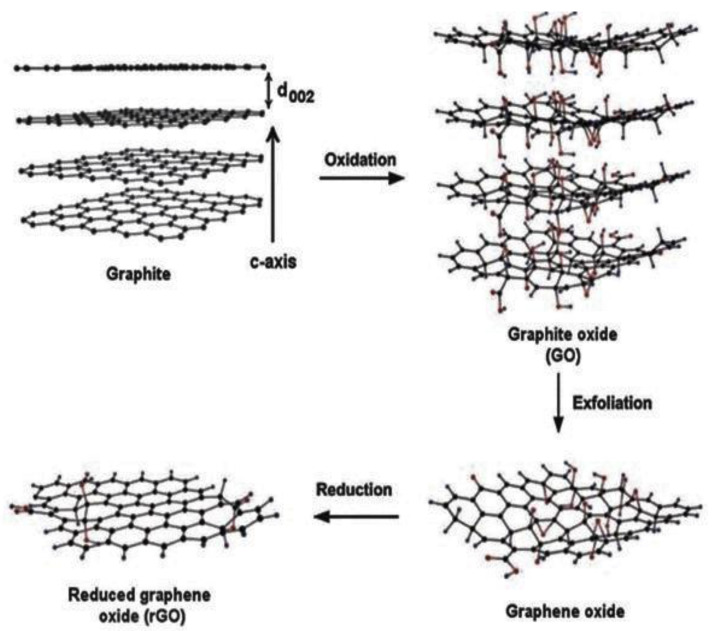
Schematic illustration of the preparation of reduced graphene oxide (rGO) from graphite. Reprinted with permission from Ref. [32]. Copyright 2011 WILEY-VCH Verlag GmbH & Co. KGaA, Weinheim.

rGO can be prepared starting from graphite, which is oxidized into graphite oxide, exfoliated, and then reduced via thermal, chemical, or electrical treatments (Scheme 4) [32]. The thermal annealing involves the reduction of GO just by heat treatment [33]. The exfoliation is triggered by the sudden expansion of CO or CO_2_ gases grown within the spaces between graphene sheets during heating. This procedure is very efficient, although it has some disadvantages: heating should be gentle to avoid explosion, while slow heating makes this reduction a time-consuming process. Besides, this approach cannot be applied for GO films on substrates such as glass and polymers, hence it is not suitable for energy applications. Other paths are microwave irradiation or photo-reduction [34], using the energy discharged by a lamp or a laser. This method can be more effective, resulting in rGO films with superior conductivity (i.e., 260 S/cm [35]), and allows the direct manufacture of electronic devices. 

Chemical reduction either at room temperature or by applying a moderate heating is one of the most important methods. The reagent more classically employed is hydrazine monohydrate (N_2_H_4_·H_2_O), which is added to a GO aqueous dispersion, resulting in agglomerated rGO nanosheets due to the increase in hydrophobicity [36]. Despite this approach possibly being appropriate for industrial production, the high toxicity of hydrazine make it unsuitable for large-scale synthesis. Consequently, other reducing agents have been suggested, such as NaBH_4_, which is very helpful at reducing C=O groups while it is less useful in the reduction of epoxy and carboxylic acid moieties [37], and cannot reduce alcohol groups. Other green reducing agents such as ascorbic acid, hydroiodic acid, and urea have been employed for GO synthesis [32]. 

On the other hand, electrochemical reduction of GO can be performed in a traditional electrochemical cell at room temperature in an aqueous buffer solution and without the need for chemical reductors. This method seems advantageous for energy and electrochemical applications.

## 3. Functionalization Procedures for the Development of Graphene/Polymer Nanocomposites 

**Scheme 5:**
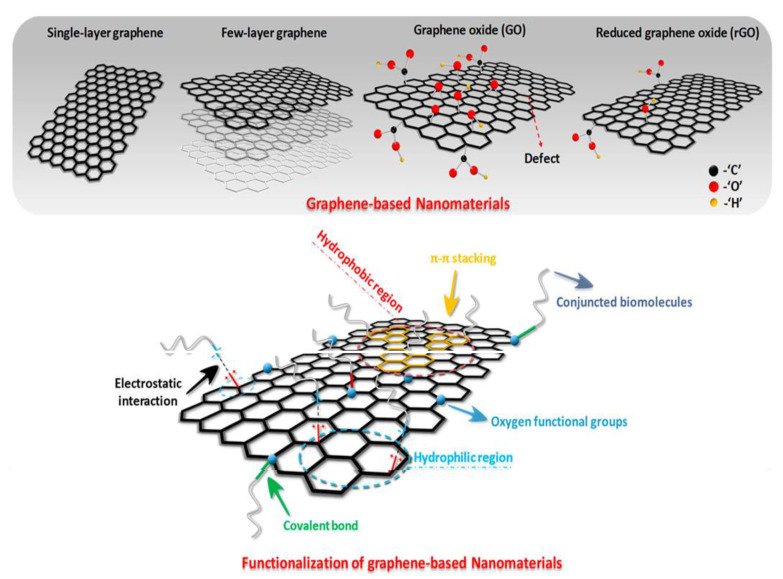
Schematic illustration of graphene-based nanomaterials, and covalent and non-covalent functionalization approaches. Reprinted from Ref. [38].

The combination of G with a polymeric matrix typically improves the polymer mechanical properties, charge dissociation, and charge transport. However, G and its derivatives require a functionalization process in order to be properly mixed with the polymer chains. A wide range of functionalization options to improve the G-polymer interaction have been reported [3,11]. These methods can be classified into two main categories: noncovalent and covalent strategies (covalent linking of polymer chains to G materials). A representative scheme of these approaches, which are described in detail in the following subsections, is shown in Scheme 5 [38]. 

### 3.1. Covalent Functionalization with Polymers 

**Scheme 6:**
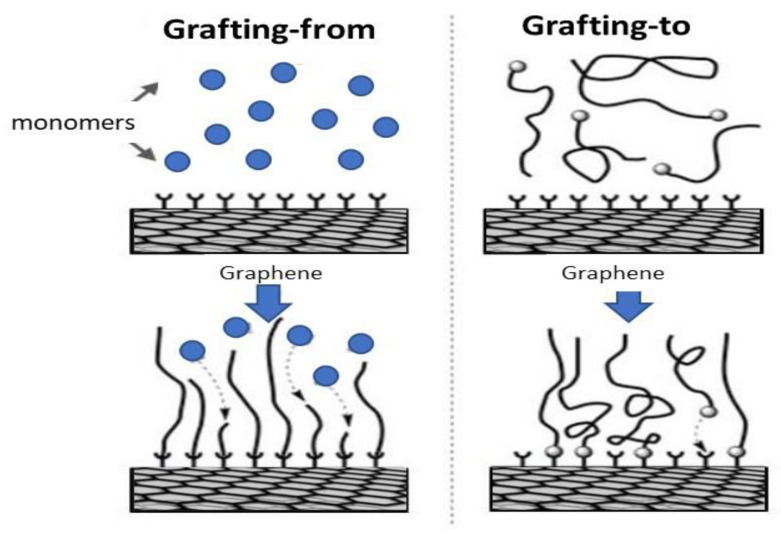
Schematic representation of the grafting-from and grafting-to approaches to functionalize graphene with polymers.

This approach involves a chemical reaction between G-based compounds and polymer chains. The irreversible linking of polymers can take place either at functional groups located on the basal planes or at the edges, and can take place in two different ways (Scheme 6): (1) Grafting-from technique, which involves the growth of the polymer directly onto the G-based compound. (2) Grafting-to technique: this pathway requires formerly prepared monomers that react with the functional groups of G-based materials. 

#### 3.1.1. Grafting-from Methods 

These are based on the anchoring of polymer-growth initiator molecules to the G-based material surface; then, the polymerization can start in these anchored initiators [39]. This technique avoids the possible steric hindrance since the polymer grows directly onto the G-based material surface, allowing the grafting of polymers with a high molecular weight, hence nanocompounds with an elevated grafting extent can be synthesized. Though, it requires a fine control of the reaction conditions as well as the amounts of initiator and substrate. Given that the initiator requires a functional group to be grafted, GO and rGO are more frequently used than G [40]. However, it is also feasible to use G if a pre-functionalization step is carried out to introduce functional groups within the structure. Several grafting-from techniques have been described in the literature, the most important being atom transfer radical polymerization (ATRP), reversible addition fragmentation chain transfer polymerization (RAFT), polycondensation, ring opening polymerization (ROP) and Ziegler–Natta polymerization. 

**Scheme 7:**
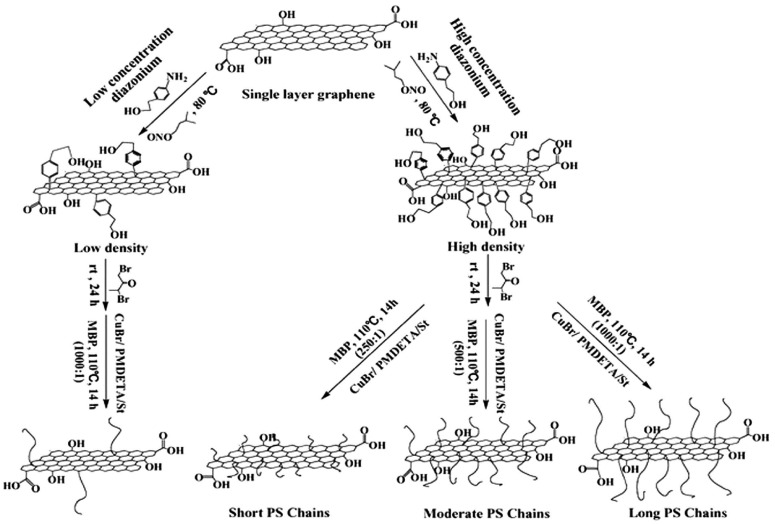
Synthetic routes for achieving controllable functionalization of polystyrene (PS)/G nanocomposites. Reprinted with permission from Ref. [42]. Copyright 1991 Royal Society of Chemistry.

The ATRP is a radical reaction that creates a carbon–carbon bond with a transition metal catalyst (a copper halide and an amine-based ligand). It comprises a fast initiation process and the development of a dynamic equilibrium between dormant and growing radicals [41]. It is suitable for the polymerization of many monomers with diverse chemical functionalities and provides good control of molecular weight, polymer composition, and structure with a low polydispersity. Though, it has some disadvantages, such as the elevated catalyst concentration needed and its removal that can be difficult and expensive, hence limiting its use on a large scale. Besides, it is an air-sensitive reaction, difficult to perform in aqueous media. For instance, it has been used for synthesizing a polystyrene (PS)/G nanocomposite [42]. rGO sheets were initially prepared via reduction of GO in the presence of a surfactant. Then, aryl diazonium salt as an ATRP initiator was covalently linked to the rGO sheets. Methyl 2-bromopropionate was used as a grafting initiator to control the chain propagation on the surfaces of graphene sheets (Scheme 7). The length and grafting density of polymer chains were tailored by changing the amount of the grafting initiator and its molar ratio with styrene monomer. Significant increases in thermal conductivity were observed for the nanocomposite with only 2.0 wt% rGO. Furthermore, the resulting PS nanocomposites with 0.9 wt% rGO showed about 70% and 57% increases in tensile strength and Young’s modulus, respectively. Additionally, it has been applied for the preparation of poly(methyl methacrylate) (PMMA) grafted from GO [43]. The initiators were immobilized by esterification reaction with the carboxylic groups of GO. In this work, the polydispersity of the grafted PMMA was approximately one, demonstrating a well-controlled process. This PMMA-g-GO displayed good solubility in organic solvents such as chloroform and methanol, and could be used as nanoreinforcement in a PMMA matrix. Due to the strong interfacial interactions between the PMMA-g-GO and PMMA, an efficient load transfer was attained, thus improving the mechanical and thermal properties of the nanocomposites. For instance, the addition of 1 wt% PMMA-g-GO led to a noteworthy improvement in the elongation at break, resulting in a more ductile and tougher material, and also increased the initial degradation temperature of the matrix by around 50 °C.

**Scheme 8:**
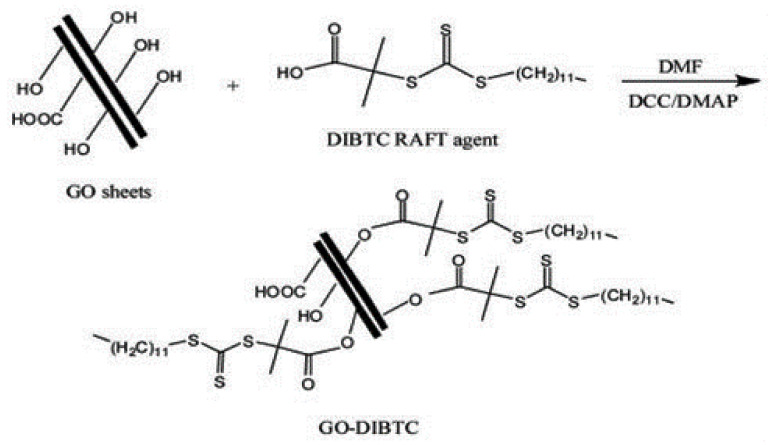
Synthesis route for the preparation of RAFT immobilized GO nanosheets. Reprinted with permission from Ref. [45]. Copyright 2011 Wiley Periodicals, Inc.

RAFT is a controlled radical polymerization reaction based on chain transfer agents, such as thiocarbonylthio (C=S) or vinyl (C=CH_2_) compounds [44], to produce low polydispersity index polymers. It works under mild conditions; polymerization can be attained by several approaches including emulsion, bulk, or suspension polymerization, and the structures are well-defined. The key benefits are that it is appropriate to varied types of monomers and that it allows controlling the polymerization of monomers soluble in water. The drawbacks are the need to select a RAFT agent for the specific polymerization and processing conditions. In this regard, PS/GO nanocomposites have been prepared via RAFT mediated mini-emulsion polymerization [45]. In this work, dodecyl isobutyric acid trithiocarbonate (DIBTC) RAFT agent was anchored to the hydroxyl groups of GO through an esterification reaction (Scheme 8). Then, stable mini-emulsions were obtained by sonicating RAFT-g-GO in styrene monomer in the presence of a surfactant, followed by polymerizing using 2,2-azobisisobutyronitrile (AIBN) as initiator to yield encapsulated PS/GO nanocomposites. The molecular weight and polydispersity of PS in the nanocomposites changed with the amount of RAFT-g-GO. The thermal stability and mechanical properties were also improved due to the intercalation of PS within the GO flakes. The storage and loss modulus of the nanocomposites with 3 and 6 wt% GO were higher than those of raw PS, while the glass transition T_g_ value decreased due to the change in the molecular weight of PS.

Polycondensation is a process in which monomers and lower molecular weight polymers react and form longer chains with higher molecular weight, while small molecules are obtained as byproducts. The resulting polymers usually present two functional groups, one in each side of the chain, because of the contribution of the functional groups of the monomers [46]. Using this procedure, GO has been functionalized with polyurethane (PU): the GO sheets reacted with 4,4′-diphenylmethane diisocyanate followed by polycondensation of poly(tetramethylene glycol) and ethylene glycol. The presence of PU chains linked to GO enhanced its dispersion within the matrix and provided a better matrix-GO load transfer, thus improving the mechanical properties, thermal stability and electrical conductivity. The tensile strength and storage modulus of PU increased by 240% and 200%, respectively, upon addition of 2.0 wt% PU-g-GO. 

**Scheme 9:**
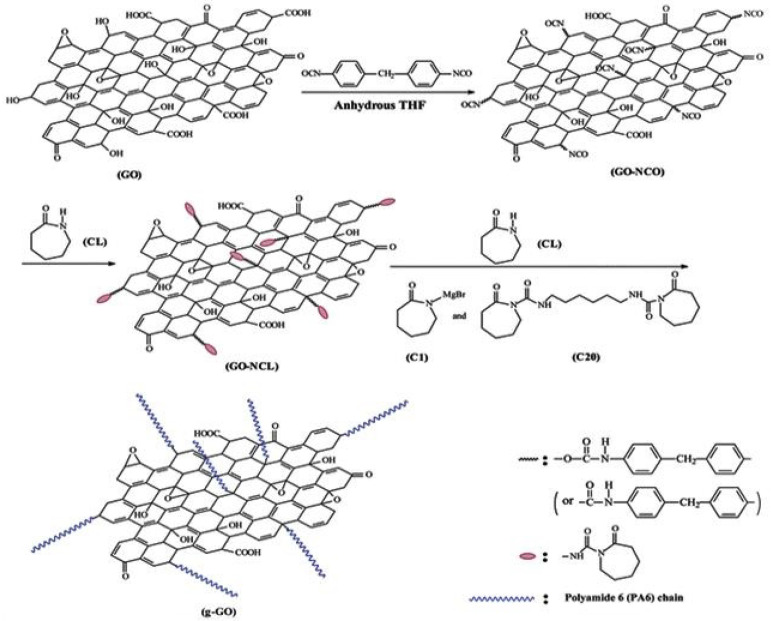
Synthesis route of polyamide 6 (PA6)-g-GO nanocomposites via anionic ring-opening polymerization. Reprinted with permission from Ref. [48]. Copyright 1991 Royal Society of Chemistry.

ROP is a method in which one termination of the polymer chain bears a group suitable to react with cyclic monomers. The end group of a compound acts as initiator and forces the opening of the first cyclic monomer, which is added to the structure as a new chain with its end group being reactive, such as the previous initiator. The polymerization continues adding more cyclic broken monomers to the structure. Different researchers also used this method for anchoring polycaprolactone (PCL) and poly(L-lactide) (PLLA) to GO [47,48]. This route was developed to prepare polyamide 6 (PA6)/GO composites (Scheme 9): ε-caprolactam (CL) was fixed onto the GO sheets coupling by 4,4′-methylenebis(phenyl isocyanate), and then PA6 was grafted from the GO surface by in situ anionic ring-opening polymerization. The polymerization was performed at 150 °C and for 20–40 min, by using a caprolactam magnesium bromide initiator (C1) in combination with a difunctional hexamethylene-1,6-dicarbamoylcaprolactam (C20) activator, leading to about 74 wt% polymer content [48]. The crystallization temperature, degree of crystallinity, and mechanical properties of PA6/GO nanocomposites increased, especially for the composites with GO loading less than 0.2 wt%, owed to the strong interfacial adhesion. This simple and effective approach can offer novel possibilities for expanding the applications of polymer-g-G nanocomposites. 

**Scheme 10:**
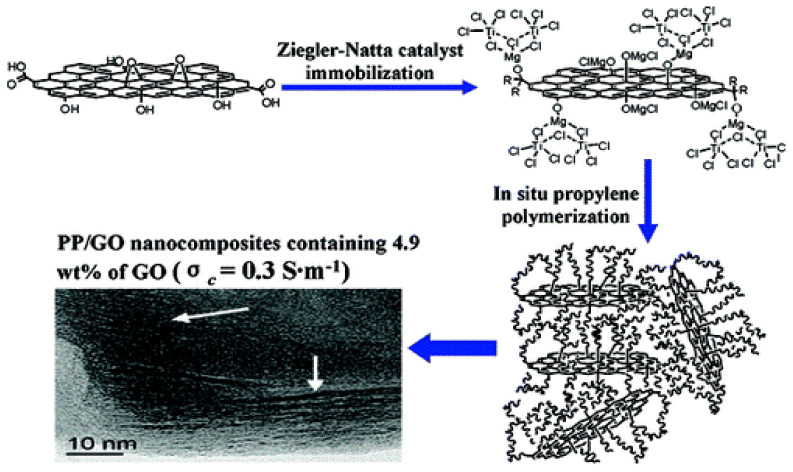
Synthesis route of polypropylene (PP)-g-GO nanocomposites via Ziegler-Natta polymerization. Reprinted with permission from Ref. [49]. Copyright 2010 American Chemical Society.

The Ziegler–Natta polymerization involves the use of catalysts to bind together vinyl-contained compounds. It usually employs TiCl_3_ or TiCl_4_, along with an aluminum-based co-catalyst [49,50], and allows synthesizing of polymers of specific tacticity. The reaction is based on the breakage of the double bound to add another molecule and continues until the termination step. As the reaction involves the functional groups of G-based compounds, the selected monomer requires a vinyl group. Polypropylene (PP)-g-GO nanocomposites have been prepared via in situ Ziegler–Natta polymerization (Scheme 10). A Mg/Ti catalyst was immobilized onto GO sheets by reacting with the surface functional groups including –OH and –COOH. Furthermore, PP polymerization was carried out together with the nanoscale exfoliation of GO. A good dispersion of the GO flakes within the PP matrix was corroborated by morphological inspection done by TEM and SEM analysis. Furthermore, high electrical conductivity was found: for instance, for a GO loading of 4.9 wt%, the conductivity was 0.3 S m^−1^. This could arise from a side reaction involving the reduction of GO sheets that takes place in one of the synthetic steps.

#### 3.1.2. Grafting-to Methods 

This approach involves the grafting of the polymer chain itself onto the G-based compound surface, although it may require former functionalization steps of either the G surface or the polymer. The goal of these functionalization steps is to provide reactive groups that can be employed for a coupling reaction. In contrast with grafting from procedures, grafting-to methods are strongly affected by steric hindrance, due to the G flakes’ size. However, they are highly versatile, since a wide variety of functional groups can be employed [51]. Besides, they allow the selection of the location of graphene, which influences the final properties. Grafting-to techniques can be divided into three types: (1) esterification/amidation reactions; (2) cycloaddition reactions; and (3) click coupling reactions.

The most extended approaches are focused on esterification or amidation reactions of the surface oxygenated groups of G with functional groups of the polymer, such as carboxylic acid or hydroxyl in order to form esters, or epoxy or carboxylic acid to form amide linkages [52]. Furthermore, other pathways to exploit the reactivity of GO and rGO have been proposed, such as the conversion of double/triple bounds using radicals (i.e., amide radicals) [53], multi-step amidation/esterification reactions (which involve a conversion of functional groups with other compounds, e.g., SOCl_2_), etc. 

For instance, functionalized rGO can react with poly(vinyl chloride) (PVC) via esterification, which was provided by a nucleophilic substitution reaction [54]. The covalent linkage of rGO to suitably functionalized PVC is an effective method to manufacture nanocomposites with enhanced thermal and mechanical properties. The addition of rGO increases the glass transition temperature of the nanocomposites, indicating chain mobility restrictions. Conjugated polymer-functionalized G-based materials have also been prepared by esterification/amidation reactions [55]. In such works, the ends of the conjugated polymers were connected to the functional groups on the graphene sheets. Thus, the solubility of the functionalized graphene in common solvents was improved, allowing solution processing. Accordingly, triphenylamine-based polyazomethine-modified GO (TPAPAM-GO) and poly(3-hexylthiophene) modified GO (P3HT-GO) were incorporated into devices via spin coating in order to obtain nanocomposites that showed non-volatile memory effect as well as higher power conversion efficiency for solar cells.

**Scheme 11:**
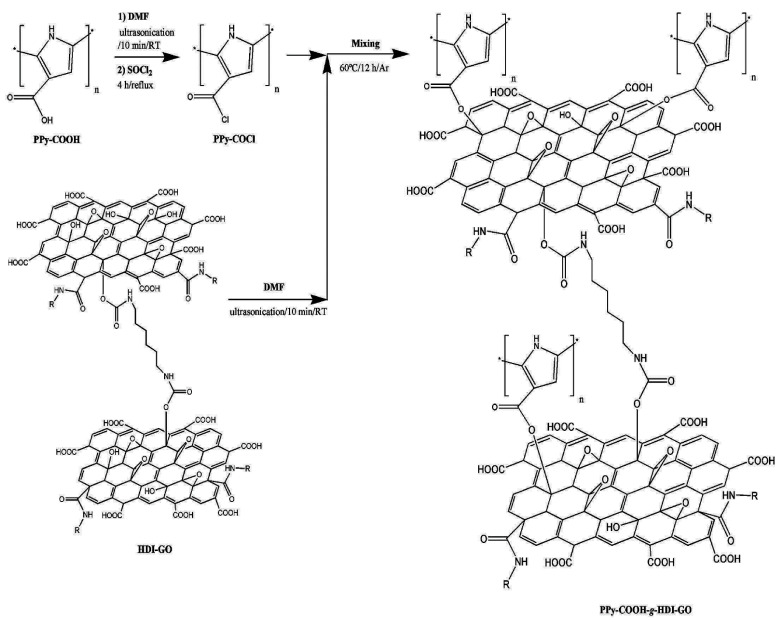
Representation of the synthesis of polypyrrole-carboxylic acid derivative (PPy-COOH) covalently anchored on hexamethylene diisocyanate (HDI)-modified graphene oxide via esterification reaction with previous functionalization of the carboxylic groups with SOCl_2_. Reprinted from Ref. [56].

Another widely used conducting polymer is polypyrrole. In this regard, a polypyrrole-carboxylic acid derivative (PPy-COOH) was grafted onto the surface of hexamethylene diisocyanate (HDI)-modified GO following two esterification approaches: activation of the carboxylic acids of the polymer by carbodiimide, and conversion of the carboxylic groups to acyl chloride. The yield of the grafting reactions (31% and 42%, respectively) was higher for the sample synthesized via formation of the acyl chloride-functionalized PPy (Scheme 11). The grafted samples showed higher thermal stability and sheet resistance than PPy-COOH. They also demonstrated better stiffness and strength, and the reinforcing efficacy was roughly retained at high temperatures. Improved mechanical performance was attained for the sample with higher grafting yield. The developed method is a useful tactic to covalently link conductive polymers onto G nanomaterials for application in flexible electronics, fuel cells, solar cells, and supercapacitors.

**Figure 1:**
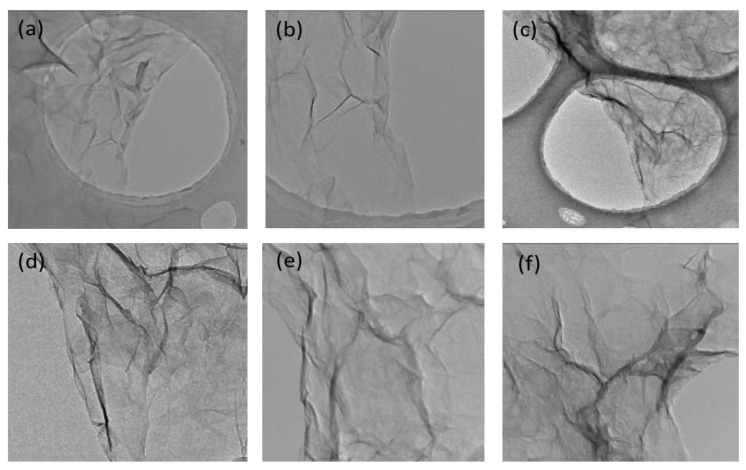
TEM images of hydroxyl functionalized G (**a**,**b**), carboxylic acid functionalized G (**c**,**d**), and G grafted PS (**e**,**f**). Reprinted with permission from Ref. [58]. Copyright 2010 American Chemical Society.

One of the most extended cycloaddition reactions with GO is the nitrene [2 + 1] [57], which requires high temperatures and azide-based polymers, such as perfluoroazides, 2,4,6-tricholoro-1,3,5-triazine combined with sodium azide, or nitrene radicals arising from other organic molecules. This strategy is simple and efficient, allowing various functional moieties (e.g., hydroxyl, carboxyl, amino, bromine, long alkyl chain, etc.) and polymers (i.e., PEG, PS) to covalently and stably anchor on GO in a one-step reaction [58], leading to individually dispersed, highly soluble, and conductive G nanosheets, as demonstrated by TEM analysis (Figure 1).

Another cycloaddition reaction approach is the Diels–Alder reaction, which relies on the formation of a cyclohexane-based derivative by a diene and a dienophile. In the case of G and its derivatives, sp^2^ C atoms can react with both dienophiles and dienes without the presence of catalyst, as described in the literature [59,60]. However, the polymer chains must comprise a diene or dienophile group within their structure, hence it could require a previous functionalization step of the polymer. 

On the other hand, the idea of using click coupling reactions to functionalize G-based compounds arises from studies with CNTs, since click reactions demonstrated good results for the functionalization of these carbon-based nanomaterials [61,62]. Click reactions were introduced by Kolb et al. in 2001 [63], and they are based on joining small units using heteroatoms as linkers. The major characteristics of click reactions are simple to perform, high yielding, wide in scope, easily-separable byproducts, high stereospecific reaction, and the possibility of using easily-handled solvents. Several reactions fulfill these requirements (e.g., cycloadditions of unsaturated species, nucleophilic ring-opening reactions, additions to C–C multiple bonds, etc.) [63], though in graphene nanocomposites with polymers, three main approaches have been used: (1) copper(I)-catalyzed alkyne-azide cycloaddition (CuAAC); (2) thiol-ene radical approach; and (3) thiol-yne radical approach.

In 2002 it was proved that CuI efficiently catalyzed azide/alkyne cycloaddition reactions [64], and many studies have been reported to date using CuAAC click reaction [65]. In the case of G-based compounds, a preceding addition of alkyne groups to the G surface is typically required, in order to react with the azide groups of the desired polymer, or vice versa. Other characteristics of CuAAC reactions are their high selectivity and that require a source of copper catalyst. Several variations of CuAAC reactions have been reported considering other metals (Ru, Ag, Au, Ni, Zn), leading to a whole family of metal-catalyzed azide-alkyne cycloaddition (MAAC) reactions.

### 3.2. Non-Covalent Functionalization with Polymers 

Three main approaches have been described among the non-covalent functionalization methods, namely, hydrogen bonding, π–π stacking, and electrostatic interactions, which do not alter the chemical structure of the G sheets, hence the electronic structure of G is preserved and its electrical conductivity is hardly affected. They are usually followed by physical methods, such as melt blending, solution mixing, or in situ polymerization [12]. They are typically simple and can be performed under mild conditions. Nevertheless, the main drawback of this method is that other components (such as surfactants) are frequently introduced. 

π–π interactions take place between the π electrons of the G basal-plane, (see Scheme 5) and the delocalized π electrons of the polymer chain chosen, which can arise from double bounds or aromatic rings [66]. Van der Waals forces and π–π stacking are of outmost importance in carbon nanostructures [67], since they promote the absorption of different molecules or the stacking of layers onto different allotropic forms of carbon. Adsorption of polymers onto the basal plane of G can be either complete—with the whole chain physisorbed—or partial—due to intrachain coiling or interchain repulsion—depending on the initial geometry, number of polymers, graphene flake size, and thickness. The planar structure of graphene is a key factor in the formation of effective π-stacking [68]. These interactions can be comparable to covalent attachment in strength and offer more stable alternatives to the weaker hydrogen bonding or electrostatic forces. Furthermore, π–π stacking also preserves the aromatic conjugation of G.

Using this approach, thermoresponsive G-polymer nanocomposites have been prepared via RAFT polymerization to synthesize a pyrene terminated poly(N-isopropylacrylamide) (PNIPAAm), followed via anchoring to G nanosheets through π–π stacking interactions [69]. The lower critical solution temperature (LCST) of pyrene-terminated PNIPAAm was found to be 33 °C. Though, subsequent to functionalization, the nanocomposites had an LCST of 24 °C, despite also being thermoresponsive in aqueous solutions. 

Hydrogen bonding is a weaker bound (2–8 kcal/mol) than π–π interactions but is widely used as non-covalent functionalization method. This bonding takes place between the oxygenated functional groups on the GO or rGO surface, and hydroxyl or carboxylic acid groups of the polymer chains. This modification of the G surface does not introduce impurities, which is nontoxic and reliable, and has important potential applications in the biomedical field [68]. This bonding is very common in the biological arena. PVA nanocomposites with GO sheets dispersed into the matrix at molecular level have been prepared [70]. The improved modulus and strength of the resulting nanocomposites were ascribed to the strong hydrogen bonding interactions between the residual oxygenated moieties of GO and the hydroxyl groups of the PVA chains. G nanocomposites with other polymers comprising hydroxyl or amine moieties such as epoxy, poly(acrylonitrile) (PAN), and polyaniline (PANI) showed unusual increments in modulus or glass transition temperature owed to H-bonding interactions [71,72].

Electrostatic interactions are another type of force employed to non-covalent functionalization. Thus, G can be non-covalently functionalized with anionic surfactants such as sodium dodecyl sulfate (SDS) solutions of different concentrations [73]. This leads to stable G dispersions due to the electrostatic repulsion between the negative charges of SDS and the π cloud of graphene. Besides, GO is soluble in water because its surface negative charge repels each other and forms a stable colloidal suspension. 

**Scheme 12:**
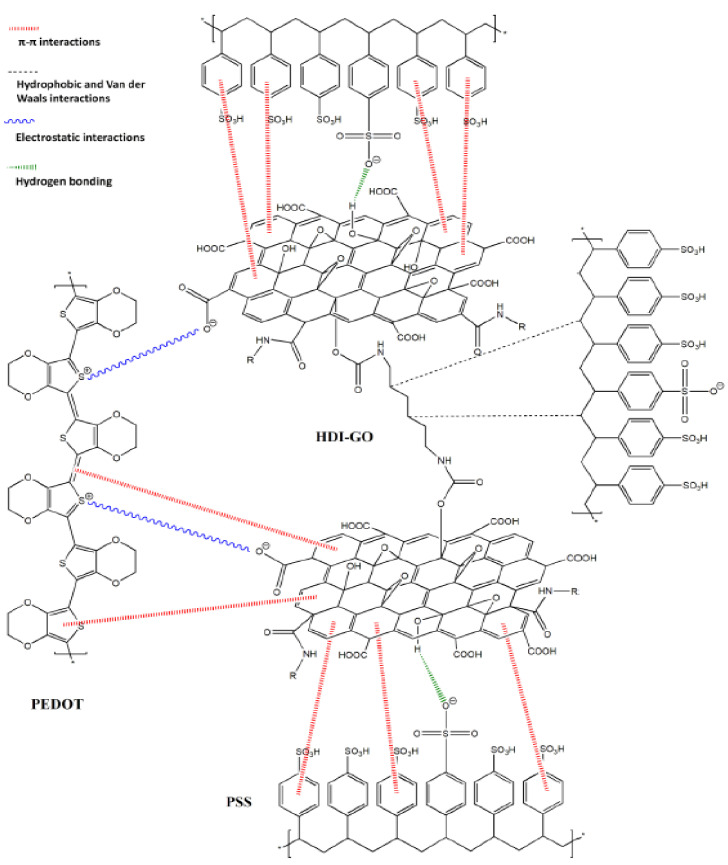
Schematic representation of the different interactions between PEDOT:PSS and hexamethylene diisocyanate (HDI)-modified GO. Reprinted with permission from Ref. [74]. Copyright 2010 American Chemical Society.

The interactions between PEDOT:PSS and hexamethylene diisocyanate (HDI)-modified GO have been investigated [74]. PEDOT chains can interact with the aromatic rings of GO and HDI-GO by π–π stacking as well as via electrostatic interactions between their negatively charged COOH groups and the positively charged PEDOT chains (Scheme 12). Moreover, their surface OH groups are disposed to interact with the negatively charged sulfonyl groups of PSS through H-bonding. In addition, HDI-GO can interact with the alkyl side chains of PSS via hydrophobic interactions and van der Waals forces. Thus, HDI-GO can interact strongly with both PEDOT and PSS chains, leading to a homogeneous dispersion within the matrix. The higher the functionalization degree of the GO, the stronger the interactions, the better the dispersion. 

**Scheme 13:**
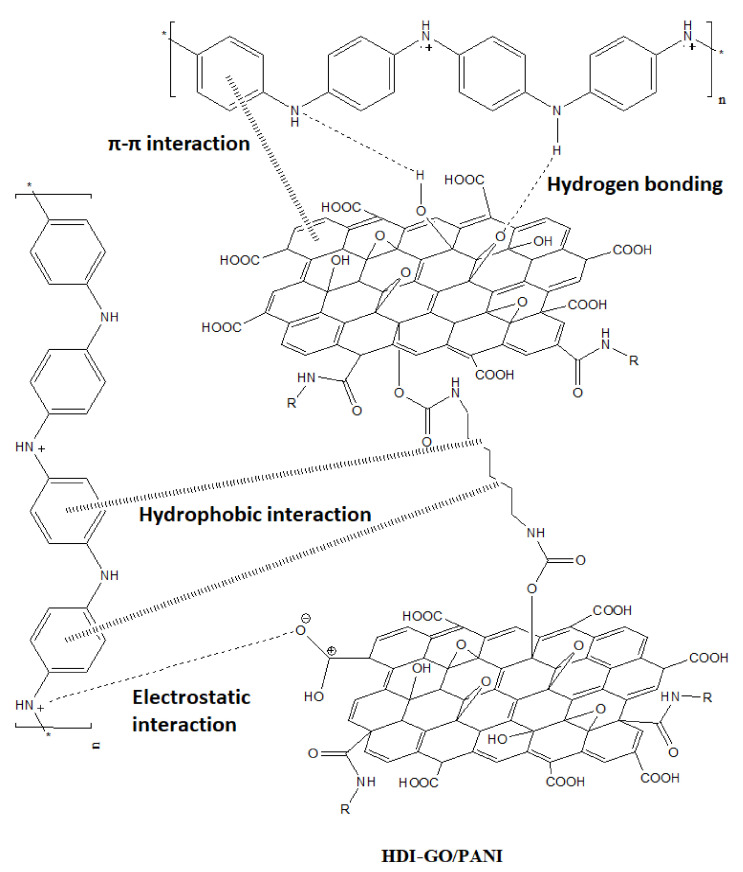
Schematic representation of the different interactions between PANI and hexamethylene diisocyanate (HDI)-modified GO. Reprinted with permission from Ref. [71]. Copyright 2010 American Chemical Society.

Similarly, different types of interactions, including H-bonding, π–π stacking, hydrophobic as well as electrostatic have been reported between PANI, in the form of emeraldine salt, and HDI-GO (Scheme 13), that result in improved interaction between the two nanocomposite components, hence very high electrical conductivity [71]. 

## 4. Applications of Graphene/Polymer Nanocomposites in the Field of Energy

**Scheme 14:**
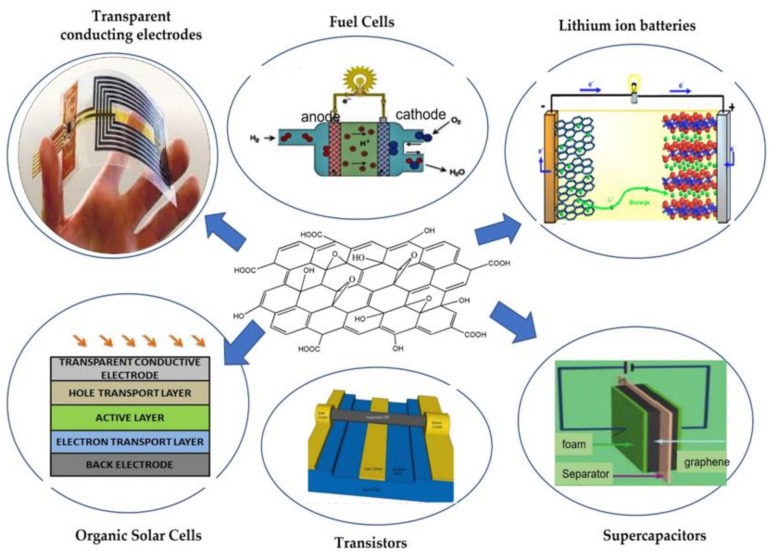
Chart flow showing potential applications of graphene-polymer composites in the field of energy.

As mentioned earlier, G/polymer nanocomposites can be developed via melt blending, solution mixing, or in situ polymerization, combined with covalent and non-covalent modification. These composites have superior optical, electrical, and thermal properties that make them suitable for a large number of applications, particularly in the field of energy. A chart flow showing these applications is shown in Scheme 14, and some of them are briefly described below. 

Lithium-ion batteries are widely used energy storage devices owed to their high energy and power density, good durability, and environmental safety. Conventional cathode materials used in these batteries are LiCoO_2_ and LiFePO_4_, although they present some drawbacks such as restricted capacity and nonrenewable resources [75]. Hence, novel materials for use as cathodes are required, including polymeric ones that present some benefits like lightness, mechanical flexibility, and easy processing. Nevertheless, polymeric cathode materials have low electrical conductivities and slow redox reactions. Thus, G-based nanomaterials can be incorporated to improve performance. For instance, PPy/rGO nanocomposites were prepared via electrodeposition on a stainless steel mesh, and their performance was compared to that of PPy/sodium p-toluenesulfonate (PPy/pTS) [76]. The nanocomposite with rGO showed improved conductivity and higher discharge capacity at low current rates, ascribed to the porous structure and the high conductivity of rGO. 

**Figure 2:**
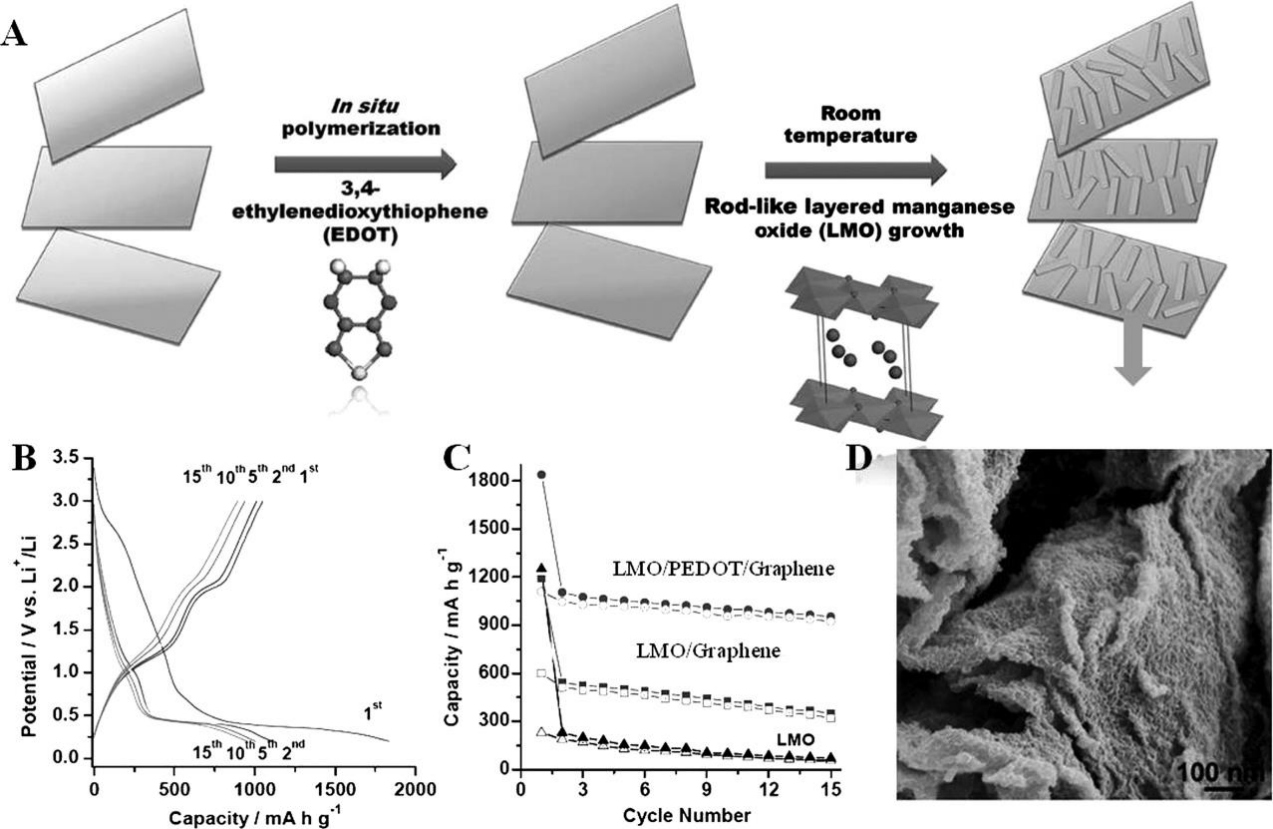
(**A**) Scheme for the preparation of LMO/PEDOT/G nanocomposite. (**B**) Capacities of the nanocomposite for the indicated charging/discharging cycles. (**C**) Change in charge/discharge capacity versus cycle number for the indicated samples. (**D**) SEM image of the LMO/PEDOT/G nanocomposite. Reprinted with permission from Ref. [77]. Copyright 2012 WILEY-VCH Verlag GmbH & Co. KGaA, Weinheim.

Layered manganese oxide (LMO) is abundant in nature, has a high theoretical capacity, and is environmentally friendly, hence is also suitable as cathode in these types of batteries. Though, its low electrical conductivity and big expansion volume throughout the charging/discharging process restricts its application. This can be solved via addition of G. Besides, polymers can act as stabilizers for developing nanostructures onto G. In particular, ternary LMO/PEDOT/G nanocomposites have been prepared via in situ polymerization of EDOT monomer in the presence of G (Figure 2A) and then used as a substrate for LMO growth [77]. A battery based on this ternary nanocomposite had improved capacity and stability over different cycles compared to those based on LMO/G binary sample or only LMO (Figure 2B,C). The improved behavior was ascribed to the presence of PEDOT/G that prevented LMO aggregation, as revealed via SEM observations (Figure 2D). 

Likewise, PANI was used to stabilize SnO_2_ bonded to rGO, and the corresponding batteries displayed high capacity and outstanding cycling stability with improved performance [78]. 

**Figure 3:**
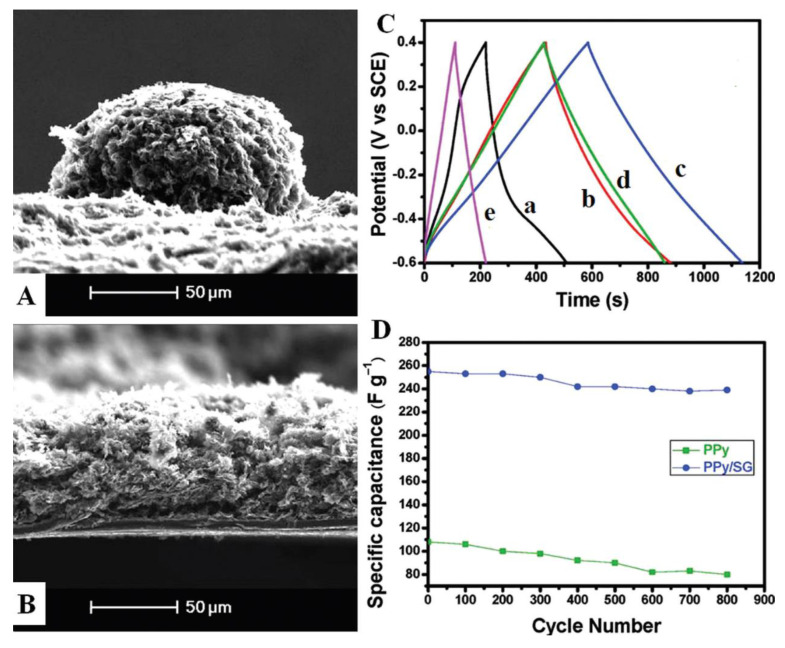
(**A**) SEM micrograph of PPy/SG nanocomposite formed at 1 C cm^−2^. (**B**) Image of the nanocomposite deposited at 2 C cm^−2^. (**C**) Charge/discharge curves of nanocomposites grown at 0.5 (a), 1.0 (b) 2 (c), and 4 C cm^−2^ (d), respectively, and (e) SG film in the aqueous solution of 1 mol L^−1^ KCl. (**D**) Cycling stability test of PPy and PPy/SG nanocomposites. Reprinted with permission from Ref. [79]. Copyright 2010 American Chemical Society.

Supercapacitors are energy storage devices that are charged/discharged at a fast speed, and should have high energy density and power density as well as long cycling life. PPy is a suitable material for use in supercapacitors. Though, the poor cycling stability and low rate of supercapacitors based on neat PPy hinder their use. In this regard, nanocomposite films based on sulphonated graphene (SG) and PPy were prepared via in situ polymerization using electrochemical deposition at 0.5 C cm^−2^ [79]. Though, semimicrospheres appeared on the film as the charge density was increaseded up to 1 C cm^−2^ (Figure 3A). When the charge density was higher than 2 C cm^−2^, the microspheres vanished and a porous film was formed (Figure 3B), which showed a high specific capacitance (Figure 3C) as well as enhanced electrochemical stability and rate performance (Figure 3D). A similar technique was used to make rGO/PPy nanocomposite, which showed about double capacitance retention than neat PPy [80]. 

Transparent conducting electrodes are a main component of optoelectronic devices. Indium tin oxide (ITO) is now the most broadly used for manufacturing this type of electrode. Though, ITO has drawbacks including some disadvantages such as elevated manufacturing costs, restricted resources, and brittleness [81]. Transparent electrodes based on rGO have been developed by solution mixing approaches, though typically displaying reduced conductivity due to contact resistance among neighboring sheets. In this regard, G-based polymer composites can be more effective. In particular, PEDOT/SG nanocomposites prepared through in situ polymerization presented higher conductivity than commercial PEDOT:PPS and transmittances higher than 80% between 400 and 1800 nm [82]. Likewise, SDBS-modified G/PEDOT:PSS nanocomposites were prepared via spin coating [83]. As the ratio of SDBS-modified G to the polymer increased, the conductivity decreased, being higher than that of commercially available ITO/polyethylene terephthalate (PET) or polyethylene naphthalate (PEN). The transparency of the nanocomposites was close to 80% at 550 nm, only slightly lower than that of ITO/PET. 

Dye-sensitized solar cells (DSSCs) have attracted a lot of interest due to their inexpensiveness, ease of fabrication, and high efficiency [81]. They comprise a working electrode of dye-sensitized titania nanocrystals, an electrolyte with a redox pair (c.a. I_2_/I_3_^−^), and a Pt counter electrode. G has been used to replace some materials of DSSCs. For instance, the use of a G film as a substitute for Pt in cells with PEDOT:PSS slightly decreases the efficiency, albeit the durability is enhanced, and the efficiency is preserved afterward for 100 cycles [84]. Moreover, liquid electrodes have been substituted by rGO with electrolyte gels to enable a faster diffusion of the I_3_^−^, hence improve efficiency [81]. 

**Figure 4:**
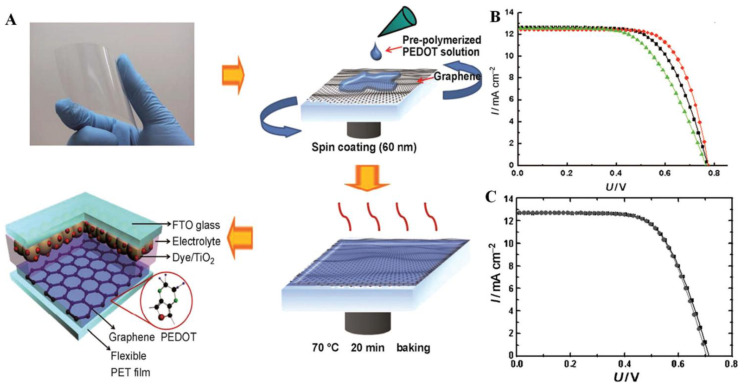
(**A**) G-coated PET and representation of the preparation process of a DSSC with a PEDOT/G/PET counter electrode. (**B**) J–V characteristics of the cells with PEDOT/G/PET (black), PEDOT/PET (green) and Pt/ITO/PET (red) as counter electrode. (**C**) J–V characteristics of bended (circles) and pristine (squares) DSSCs using a PEDOT/G/PET counter electrode. Reprinted with permission from Ref. [85]. Copyright 2012 WILEY-VCH Verlag GmbH & Co. KGaA, Weinheim.

Polymeric nanocomposites with G have been tested as flexible counter electrodes of DSSCs. Thus, a PEDOT layer was spin coated onto a G-coated PET film (Figure 4A), and the cell with this PEDOT/G/PET counter electrode had an efficiency of 6.3%, close to that with Pt/ITO and higher than that with PEDOT counter electrode (Figure 4B) [85]. Moreover, the cell with PEDOT/G/PET has a high fill factor and outstanding performance even after bending (Figure 4C). G/polymer nanocomposites have also been used in organic solar cells (OSCs). The first use of G as an electron acceptor material in these types of cells was reported in 2008 [86]. A film of poly(3-octylthiophene) (P3OT) as donor and phenyl isocyanate-functionalized G as acceptor was prepared by spin coating. The π–π interactions between P3OT and functionalized G made this nanocomposite effective as the active layer in OSCs, though the efficiency was low (~1.4% for a G loading of 5 wt%). The annealing process detached some functional groups, thus enhancing the charge transport, and increased the matrix crystallinity, and hence the cell efficiency. Besides, PEDOT:PSS/GO nanocomposites have been used as hole transport layer in OSCs, leading to an efficiency of 4.3%, better than that of a cell with only PEDOT:PSS (3.6%), as well as in improved durability and reproducibility, ascribed to the well complemented work function between GO and PEDOT:PSS that increases charge mobility [87]. In addition, GO blocks electrons, and solves the acid corrosion problems in the ITO layer produced by PEDOT:PSS, thus prolonging cell life. 

## 5. Conclusions and Future Perspectives

Graphene is a novel nanomaterial for electrical and optical devices owed to its atom-thick 2D structure, large specific surface area, outstanding electrical and thermal conductivity, superior mechanical stiffness combined with flexibility, and high optical transparency. However, G-based materials, including GO and rGO, are frequently blended with polymers in order to form nanocomposites with enhanced processibility, mechanical, chemical, and electrochemical properties due to synergistic effects. In this review, recent advances in the development of polymer/G nanocomposites were summarized. G and its derivatives typically require a functionalization process of being properly mixed with the polymer chains. These functionalization methods, namely, noncovalent and covalent strategies, were reviewed and selected examples were described. Furthermore, the applications of these polymeric nanocomposites in the field of energy were highlighted. When G is blended with conducting polymers, it offers mechanical support to the matrix, and thus enhances the cycling performance and the capacitance. 

Although much progress has been attained to date, the research in this arena is still in its initial stage, and several problems remain unsolved to reach their full potentials: Firstly, G used to develop polymer nanocomposites typically has considerably lower conductivity than that reported for a perfect graphene monolayer. Thus, the thermal and electrical conductivities of the nanocomposites are much lower than the predictions, and do not fulfill the requirements for certain applications such as transparent conductive electrodes or counter electrodes of OSCs. Secondly, a method that allows the synthesis at a large scale and at low cost of high-quality graphene is immediately required, without compromising the G nanostructure owed to sheet restacking or aggregation. Numerous efforts have already been dedicated to solve this issue, such as the fabrication of G sheets with large lateral dimensions, albeit only very low efficiencies have been attained. Thirdly, the real specific surface area of G and its derivatives in the polymer nanocomposites are much lower than the predictions owed to the strong π–π stacking among G sheets. This issue becomes even worse when blending with polymers. The mixing can be carried out via three main methods: solution blending, in situ polymerization, and melt blending. Melt blending is a promising approach because of its versatility, compatibility with current processing technologies, and environmental safeness due to the absence of solvents, but some polymers lack stability at high temperatures. Thus, novel techniques that preserve the large specific surface area during G dispersion within polymer matrices, able to tailor the microstructure, are required. Processing approaches such as electrospinning or layer-by-layer assembly provide effective means to control the morphology at the nanometer scale and enable the integration of considerably higher nanofiller content compared to conventional techniques, but have low potential at an industrial level owed to their low manufacture speed. Besides, the use of G-based polymeric composites in solar cells is still at an early stage, and their performance is still far from those of their traditional counterparts. Thus, the compositions and morphologies of the nanocomposites require additional optimization. All these aspects should be taken into account to obtain a deeper understanding of the structure-property relationship and designs of graphene/polymer nanocomposites. Overall, these types of nanocomposites have great potential in a wide number of applications, particularly in the field of energy, and will have important applications when commercialized at a large scale in the near future.
